# Right ventricular function declines prior to left ventricular ejection fraction in hypertrophic cardiomyopathy

**DOI:** 10.1186/s12968-022-00868-y

**Published:** 2022-06-13

**Authors:** Masliza Mahmod, Betty Raman, Kenneth Chan, Sanjay Sivalokanathan, Robert W. Smillie, Azlan H. Abd Samat, Rina Ariga, Sairia Dass, Elizabeth Ormondroyd, Hugh Watkins, Stefan Neubauer

**Affiliations:** 1 Division of Cardiovascular Medicine, Radcliffe Department of Medicine, University of Oxford Centre for Clinical Magnetic Resonance Research (OCMR), University of Oxford, John Radcliffe Hospital, Headley Way, Oxford, OX3 9DU UK; 2 Division of Cardiovascular Medicine, Radcliffe Department of Medicine, University of Oxford, Oxford, UK

**Keywords:** Hypertrophic cardiomyopathy, Cardiac magnetic resonance, Right ventricular function

## Abstract

**Background:**

The right ventricle (RV) in hypertrophic cardiomyopathy (HCM) tends to be neglected, as previous efforts have predominantly focused on examining the prognostic value of left ventricular (LV) abnormalities. The objectives of this study were to assess RV function in HCM, changes over time, and association with clinical outcomes.

**Methods:**

Two hundred and ninety HCM patients with preserved LV ejection fraction (LVEF ≥ 55%) and 30 age- and sex-matched controls underwent cardiovascular magnetic resonance (CMR). All patients were followed up for clinical events for a median duration of 4.4 years. Sixty-three patients had a follow-up CMR undertaken at a median interval of 5.4 years. Main study measures and outcomes were RV function (RV ejection fraction (RVEF) and RV strain) at baseline, temporal changes in RV function over time and prognostic value of RV dysfunction for predicting cardiovascular outcomes in HCM.

**Results:**

When compared to controls, HCM patients exhibited lower RV and LV peak global longitudinal systolic strains on feature-tracking analysis of cine images, while RVEF and LVEF were within the normal range. On follow-up CMR, both RV and LV strain parameters decreased over time. RVEF decreased at follow-up (65 ± 7% to 62 ± 7%, P < 0.001) but the change in LVEF was not significant (68 ± 10% to 66 ± 8%, P = 0.30). On clinical follow up, reduced RVEF was an independent predictor of non-sustained ventricular tachycardia (NSVT) [HR 1.10 (95% CI 1.06–1.15), P < 0.001] and composite cardiovascular events (NSVT, stroke, heart failure hospitalisation and cardiovascular death) [HR 1.07 (95% CI 1.03–1.10), P < 0.001]. RV longitudinal strain was an independent predictor of NSVT [HR 1.05 (95% CI 1.01–1.09), P = 0.029]. Patients with RVEF < 55% showed an increased risk of NSVT and composite cardiovascular events. In contrast, LVEF and LV global longitudinal strain were not predictive of such events on multivariable analysis.

**Conclusions:**

In HCM, RV function, including RV strain, and LV strain decrease over time despite preserved LVEF. Reduction in RV but not LV function is associated with adverse cardiovascular outcomes. Assessing RV function in early HCM disease might have a role in risk stratification to prevent future cardiovascular events.

**Supplementary Information:**

The online version contains supplementary material available at 10.1186/s12968-022-00868-y.

## Background

Hypertrophic cardiomyopathy (HCM) is the most common genetic cardiac disease with an estimated prevalence of 1 in 500 [1]. While the majority of patients are asymptomatic, a significant subset experience life-threatening arrhythmias, heart failure (HF) or stroke [2–4]. Identifying those at risk of adverse cardiovascular outcomes has been a research priority for over six decades. For years, the left side of the heart in HCM has been extensively studied, with the extent of hypertrophy, left atrial (LA) size and left ventricular (LV) outflow obstruction forming integral parts of contemporary risk stratification guidelines [1, 5]. In contrast, the role of the right ventricle (RV) in risk stratification of HCM remains poorly understood. Recently, a number of studies have reported abnormalities involving the RV in HCM patients including reduced RV systolic and diastolic function [6, 7], reduced end-diastolic volume [8] and increased wall thickness [9].

RV dysfunction has been shown to predict adverse outcomes in a number of other cardiovascular diseases. For example, in a study of HF patients with preserved LV ejection fraction (HFpEF) [10], RV function was an independent predictor of cardiovascular mortality. Similarly, in patients with dilated cardiomyopathy, Gulati et al. demonstrated that RV function was an independent determinant of worse prognosis [11]. Whether or not RV function can discriminate risks in HCM patients despite normal LV function is unknown.

RV functional assessment has improved considerably with the use of cardiovascular magnetic resonance (CMR) in clinical practice. When compared to echocardiography, CMR provides comprehensive views of the RV at high resolution with superior blood-myocardium discrimination. In addition, advanced post-processing analysis of cine-images such as quantitative myocardial strain analysis has permitted further characterisation of RV function [12].

In this study, we sought to characterise RV function comprehensively using CMR (RV ejection fraction (RVEF) and RV strain) in HCM patients with preserved LV function. We further examined the temporal changes in RV function over time and investigated the prognostic value of RV dysfunction for predicting cardiovascular outcomes in HCM.

## Methods

### Study population and protocol

HCM patients aged > 18 years, without LV impairment were enrolled from the Inherited Cardiac Conditions (ICC) clinic at the Oxford University Hospitals NHS Foundation Trust (2003–2016).These patients had either a genetic diagnosis of HCM and LV wall thickness of ≥ 13 mm, or non-familial HCM patients with a LV wall thickness ≥ 15 mm, but no other cause of hypertrophy identified [5]. Genetic diagnosis of HCM was made based on 13 genes associated with HCM (MYBPC3: myosin binding protein C; MYH7: myosin heavy chain; TNNI3: cardiac troponin I; TNNT2: cardiac troponin T; MYL2: regulatory myosin light chain; MYL3: essential myosin light chain; TPM1: alpha tropomyosin; ACTC1: cardiac actin; CSRP3: muscle LIM protein; PRKAG2: AMPK γ2; PLN: phospholamban; GLA: alpha galactosidase; FHL1: four and a half LIM domains 1). Genotype positive patients without LVH were excluded. Patients with a pacemaker/implanted cardiodefibrillator (ICD) were excluded as it was a contraindication to CMR. All patients had an echocardiogram as part of routine clinical care. Those with impaired LV function (LV ejection fraction (LVEF) < 55%), uncontrolled systemic hypertension (defined by ≥ 2 antihypertensive agents or ambulatory blood pressure ≥ 140/90 mmHg), pulmonary hypertension (pulmonary artery systolic pressure > 38 mm Hg on echocardiogram), severe primary valvular heart disease, and ischaemic heart disease were excluded. Age- and sex-matched healthy controls were recruited for comparison. They were recruited from the local population identified by word of mouth, poster advertisements around hospital and university. Healthy controls were included if they were 18 years or older, asymptomatic without known medical conditions such as heart disease, hypertension, diabetes, previous stroke and not on any medications. Frequency matching was performed based on the age and gender for the HCM cohort. All study participants gave informed consent to participate in the study, which was approved by the Local Research Ethics Committee 09/H0604/110).

### Cardiac magnetic resonance

**Fig. 1:**
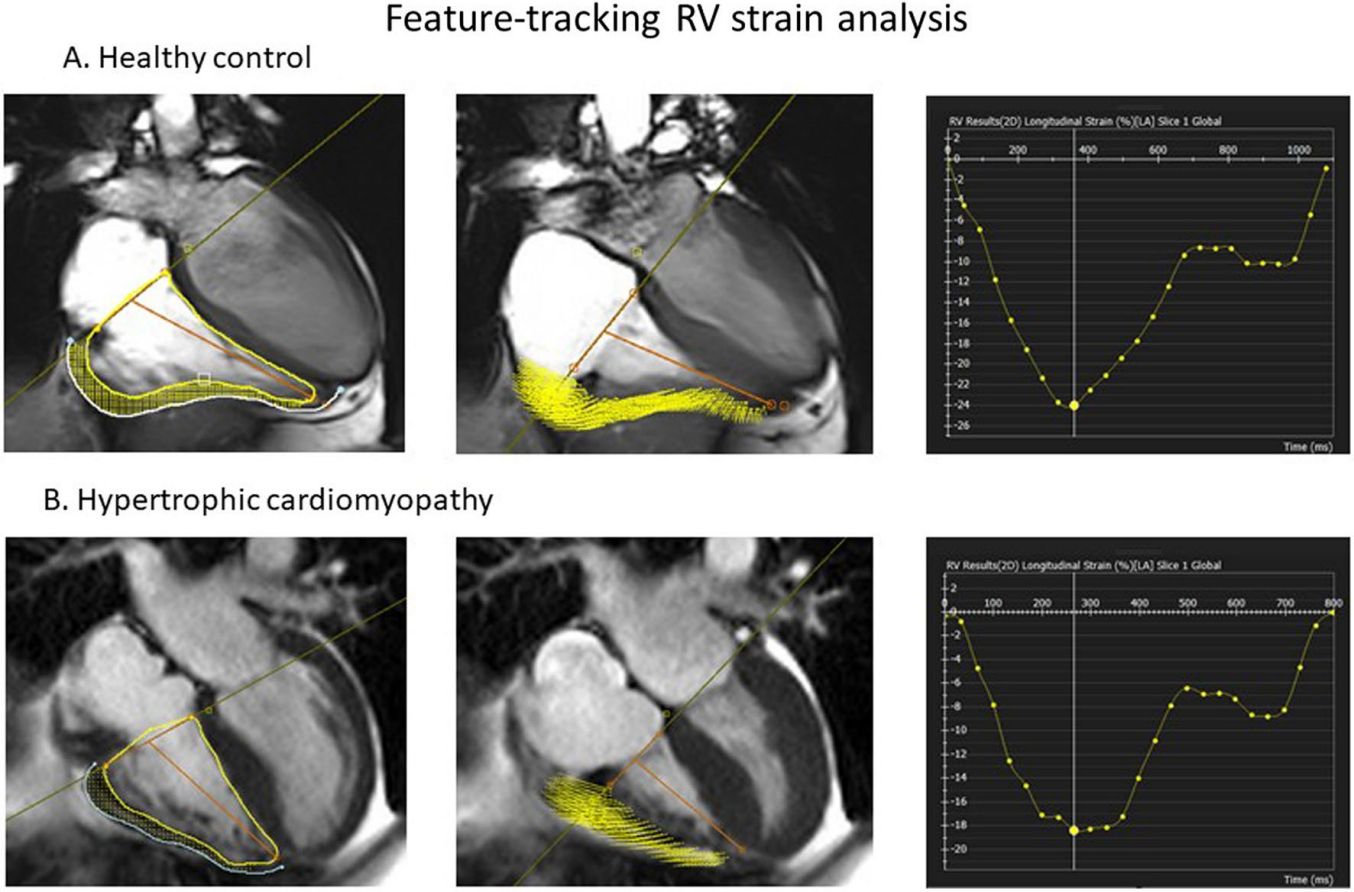
Example of cardiovascular magnetic resonance (CMR) feature tracking (FT) of right ventricle (RV). **A** Healthy control and **B** hypertrophic cardiomyopathy (HCM) throughout cardiac cycle (diastole, left column and systole, middle column). The right column shows global peak longitudinal strain curves

**Fig. 2:**
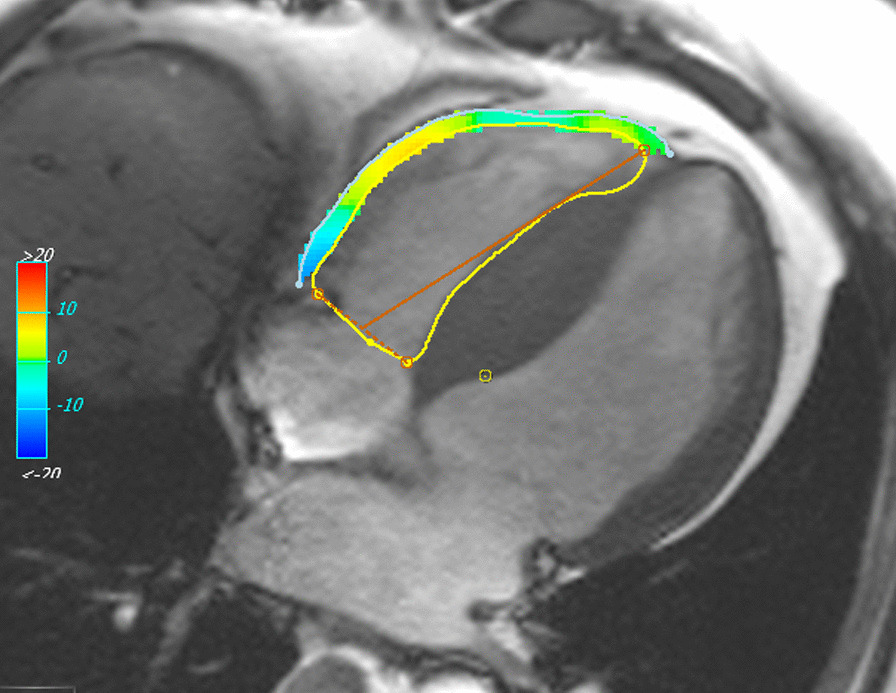
A movie of RV feature tracking in horizontal axis view demonstrating RV contours corresponding to the RV anatomy

All patients underwent CMR imaging at 1.5 T or 3 T CMR scanners (Avanto and TIM-Trio, Siemens Healthineers, Erlangen, Germany). Cine imaging was performed using standard methods, acquiring images in the short axis (SAx), axial and horizontal long axis (HLA) planes [13]. RV volumes and RVEF were determined by contouring RV endocardium both at end-diastole and end-systole of the whole heart short-axis cine images. RV wall thickness was measured from the RV free wall in the short axis view. Right ventricular hypertrophy was defined in cases where RV free wall thickness of ≥ 8 mm [9]. RV longitudinal strain was obtained from horizontal long-axis view, excluding the septum (RV free wall strain – RVFWS). Endocardial and epicardial contours were drawn from the tricuspid valve anulus to the apex of the RV and back to the opposite annulus and segmented respectively. Circumferential and radial strains were derived from SAx cines. Contours were tracked automatically over the entire cardiac cycle to derive strain results [14, 15]. For LV strain, segmentation was performed including the ventricular septum as previously established [16, 17]. A less negative (i.e. more positive) strain value means a “decrease” in strain. High resolution images were obtained as shown as examples in Figs. 1 and 2 (Additional file 1: movie). Inter- and intra- observer reproducibility for RVEF and strain measurements were determined by intraclass correlation coefficients on 30 randomly selected cases (Additional file 2: details in the supplement) and were very good. The measurements were performed in cvi42 (Circle Cardiovascular Imaging, Calgary, Alberta, Canada) blinded to the clinical information and outcomes. Late gadolinium enhancement (LGE) was considered to be present when the area of contrast enhancement could be seen visually by an experience CMR reader. LGE quantification was performed for LV but not RV, using cvi42 (Circle Cardiovascular Imaging). The threshold of 6 standard deviation (SD) above remote myocardium technique was used to quantify fibrosis as previously described [18]. Fibrosis volume was expressed as LGE mass (g).

### Patient follow up

HCM patients were followed up with (1) ICC clinic visits at regular intervals of 6 months to 1 year, (2) hospital records for encounters with health services and (3) telephone interview for cases that were not followed up in Oxford. Follow up CMR scans were done as part of an ethically approved prospective research study (reference: 12/LO/1979). Funding to support follow-up scans (for longitudinal assessment) were only available for 63 patients. Patients were invited randomly by a clinical research fellow who had no prior knowledge of disease severity or symptoms status. All patients were part of a genetic research database Progeny ICC and had agreed to be contacted for future research. Clinical events during the follow-up period were recorded, and arrhythmic events were verified by reviewing 24-h Holter reports. Clinical events included new onset atrial fibrillation (AF), ≥ 3 beats of non-sustained ventricular tachycardia (NSVT) at a rate of ≥ 120 beats/min, stroke or embolic event, HF outcomes which included HF hospitalisation and progression to New York Heart Association (NYHA) class III/IV, and all-cause mortality. The combination of incident AF, NSVT, HF outcome, and cardiovascular death was used as composite cardiac end-point.

### Statistical analysis

Categorical variables are summarised as proportions (%) and analysed using chi-square test where appropriate. Continuous variables are presented as means ± standard deviation for normally distributed data. Variable were tested for normality with Kolmogorov–Smirnov statistics for normal distribution. Student t-test was used for comparisons of quantitative measurements between HCM and healthy controls. Paired t-test was used to analyse interval changes in CMR measurements. Pearson correlation analysis was used to study the relationships between RVEF and strain results. Outcome and survival data were analysed using Cox proportional hazards regression model and presented as Kaplan–Meier curves. Univariate and multivariable binary Cox regression model was used to estimate the hazard ratio (HR) for RVEF, RV strain and LVEF, LV strain (Additional file 2: supplementary material) as continuous variables. RVEF and LVEF were made negative to permit comparison of HR for ejection fractions and strain parameters.

The variables included in each of the multivariable models were univariate predictors for each of the pre-specified outcome (Additional file 2: supplementary material). In places where two variables were highly correlated (e.g., LV mass versus wall thickness), the variable with the highest hazard ratio from univariate table was used and adjusted for in the multivariable model to avoid for collinearity (Additional file 2: Supplementary Tables 1–4). In the case of NSVT, we adjusted for age, syncope, LA diameter (by CMR), medication use, RV mass, LV longitudinal strain, LV maximum wall thickness and LGE presence. For AF, the multivariable model was adjusted for age. For heart failure outcomes, we adjusted for age, gender, body mass index, NYHA class, RV mass index, LV end diastolic volume, maximum LV wall thickness, LGE, LV radial strain and crista supraventricularis. For composite cardiovascular events, the model was adjusted for univariate predictors which included age, body mass index (BMI), LA diameter, NYHA class, medication use, maximum LV wall thickness, family history of sudden cardiac death, LVEF fraction, LV mass index, LGE mass and crista supraventricularis. RV crista supraventricularis is a prominent hypertrophied part of the RV muscular structures. In some patients with HCM, the crista supraventricularis is not only significantly hypertrophied but inserts directly adjacent to the ventricular septum. As a result, this RV muscle structure may be inappropriately included in the measurement of septal thickness-resulting in an overestimation of the maximal LV wall thickness. Maron et al. found a close association between the size of RV crista supraventricularis and RV wall thickness and speculated that this structure may contribute to additional risks [9].

Univariate Cox regression was further undertaken for binary variables: RVEF < 55% and RV strain > − 21.3%. The RVEF cut off of < 55% was chosen based on previous literature [13, 19] and RV strain was separated by median value. Relative risks were presented as HR with 95% confidence intervals (CI). All multivariable analyses were adjusted for corresponding univariate predictors (Additional file 2: details in the supplement). All statistical analyses were performed with SPSS (version 24.0, Statistical Package for the Social Sciences, International Business Machines, Inc., Armonk, New York, USA), and GraphPad Prism (version 7.0, Graph-Pad Software, La Jolla, California, USA). Sample size calculation was based on prior knowledge of event rates in HCM population (Additional file 2: details in the supplement). For power analysis based on hazard ratio, post hoc analysis revealed that given an event rate (NSVT) of 12% in HCM patients with RVEF ≥ 50% and 40% in patients with RVEF of < 50%, a sample size of > 237 gave the study power of > 80% and α of 0.05, to detect a significant difference in hazards between the two groups.

## Results

### Baseline characteristics

The baseline characteristics of 290 HCM patients (52 ± 15 years, 74% male) and 30 healthy controls (54 ± 15 years, 77% male) are summarised in Table 1. One hundred and forty eight (51%) of patients were on beta-blockers. Fifty-two (17%) of patients had a resting LV outflow tract gradient of > 30 mmHg. The average 5 year risk of sudden cardiac death (SCD) based on European Society of Cardiology guidelines (ESC) (2.2 ± 1.6%) [5]. Of 290 patients, 241 patients had genetic testing, of whom 84 had positive tests, 129 had negative tests and 28 had VUS (variant of uncertain significance). Echocardiography showed mildly dilated LA in the HCM cohort (LA volume 66 ± 26 ml). There was no evidence of moderate or severe tricuspid regurgitation or pulmonary hypertension. The mean tricuspid regurgitation gradient was 22 ± 8 mmHg and all patients enrolled had normal pulmonary artery systolic pressure (≤ 36 mmHg).

**Table 1:** Baseline characteristics and CMR results of HCM patients and healthy controls

	HCM (n = 290)	Healthy controls (n = 30)	P-value
Age (years)	52 ± 15	54 ± 15	0.70
Male, n (%)	215 (74%)	23 (77%)	0.83
Body mass index (kg/m.^2^)	28 ± 5	26 ± 7	0.12
Clinical history, n (%)			
Smoking	52 (17%)	−	
Hypertension	86 (30%)	−	
Diabetes	22.0 (78%)	−	
Atrial fibrillation	18 (6%)	−	
LVOT gradient > 30 mmHg	52 (17%)		
NYHA class I, II, III, IV (%)	223, 47, 19,1 (77, 16, 7, 1)	–	
ACC/AHA SCD Major Risk (0, 1, 2, 3)	200, 73, 16, 1 (69,25,6,1)		
ESC 5 year SCD risk	2.2 ± 1.6		
Medications, n (%)			
Aspirin	91 (31%)	−	
ACE-I/ARB	68 (23%)	−	
Beta-blockers	148 (51%)	−	
Calcium channel blockers	64 (22%)	−	
Diuretics	10 (3%)	−	
Amiodarone	18 (6%)	−	
Digoxin	47 (12%)	−	
Oral anticoagulants	26 (8%)	−	
CMR results			
RVEDV (ml)	152 ± 42	162 ± 44	0.70
RVESV (ml)	56 ± 20	55 ± 20	0.77
RVSV (ml)	96 ± 27	107 ± 27	0.04
RVEF (%)	63 ± 7	66 ± 6	0.02
RV mass (g)	38 ± 11	26 ± 75	< 0.001
RV mass index (g/m.^2^)	19 ± 4	14 ± 3	< 0.001
Maximum RV wall thickness	6.7 ± 1.7	3.9 ± 0.8	< 0.001
RV LGE present	8 (3%)	0	–
Crista supraventricularis	32 (11%)	–	–
RV peak radial strain (%)	19.3 ± 6.4	23.8 ± 6.8	0.004
RV peak circumferential strain (%)	− 11.9 ± 3.2	− 14.2 ± 3.4	< 0.001
RV peak longitudinal strain (%)	− 19.5 ± 4.1	− 22.1 ± 3.0	0.001
LGE present, n	23 (77%)	0	–
LGE mass, g	17 (IQR 7–26)	–	–
LVEDV (ml)	144 ± 37	153 ± 39	0.21
LVESV (ml)	45 ± 16	50 ± 14	0.067
LVSV (ml)	100 ± 25	97 ± 26	0.51
LVEF (%)	70 ± 6	67 ± 5	0.03
LV mass (g)	160 ± 52	106 ± 24	< 0.001
LV mass index (g/m^2^)	80 ± 23	55 ± 11	< 0.001
Maximal LV wall thickness (mm)	20.0 ± 4.2	9.7 ± 1.8	< 0.001
LV peak radial strain (%)	34.7 ± 11.48	36.7 ± 6.4	< 0.001
LV peak circumferential strain (%)	−15.3 ± 4.2	−20.5 ± 2.5	< 0.001
LV peak longitudinal strain (%)	−18.1 ± 3.8	−19.2 ± 1.9	0.009

ACE-I/ARB, Angiotensin converting enzyme inhibitor/Angiotensin receptor blocker; ACC American College of Cardiology; ESC, European Society of Cardiology; SCD, sudden cardiac death; ICD, Implantable cardiac defibrillator. LV, left ventricular; LVEDV, left ventricular end-diastolic volume; LVESV, left ventricular end-systolic volume; LVEF, left ventricular ejection fraction, LVOT, left ventricular outflow tract; LVSV, left ventricular stroke volume; NYHA, New York Heart Association; RVEDV, right ventricular end-diastolic volume; RVESV, right ventricular end-systolic volume; RVEF, right ventricular ejection fraction; RVSV, right ventricular stroke volume

### Assessment of LV and RV function in HCM

CMR results are shown in Table 1. As expected, LV mass, LVEF and maximum wall thickness were significantly higher in HCM when compared to controls. LV LGE was present in 77% of HCM patients. Similarly, RV mass and wall thickness were increased in HCM, but RV end-diastolic volumes were similar to healthy controls. RV stroke volume was reduced in HCM. RV hypertrophy was seen in 64 (22%) patients and RV LGE was seen in only 8 (3%) of HCM patients. None of the healthy controls had evidence of RV hypertrophy or LGE. Crista supraventricularis was seen in 32 (11%) of HCM patients. HCM patients had higher LVEF while RVEF was only mildly reduced (63 ± 7%) compared to healthy controls (66 ± 6%, P = 0.02). Consistent with previous reports [19], RVEF positively correlated with LVEF (r = 0.36, P < 0.001). RV strain assessment showed a significant reduction in all RV systolic strain parameters compared to healthy controls (Table 1). There was modest correlation r = 0.25, P < 0.001, between RVEF and RV longitudinal strain. Figure 1 shows an example of abnormal peak RV longitudinal strain on CMR in HCM compared to healthy control.

### Progression of RV dysfunction on follow-up CMR

**Fig. 3:**
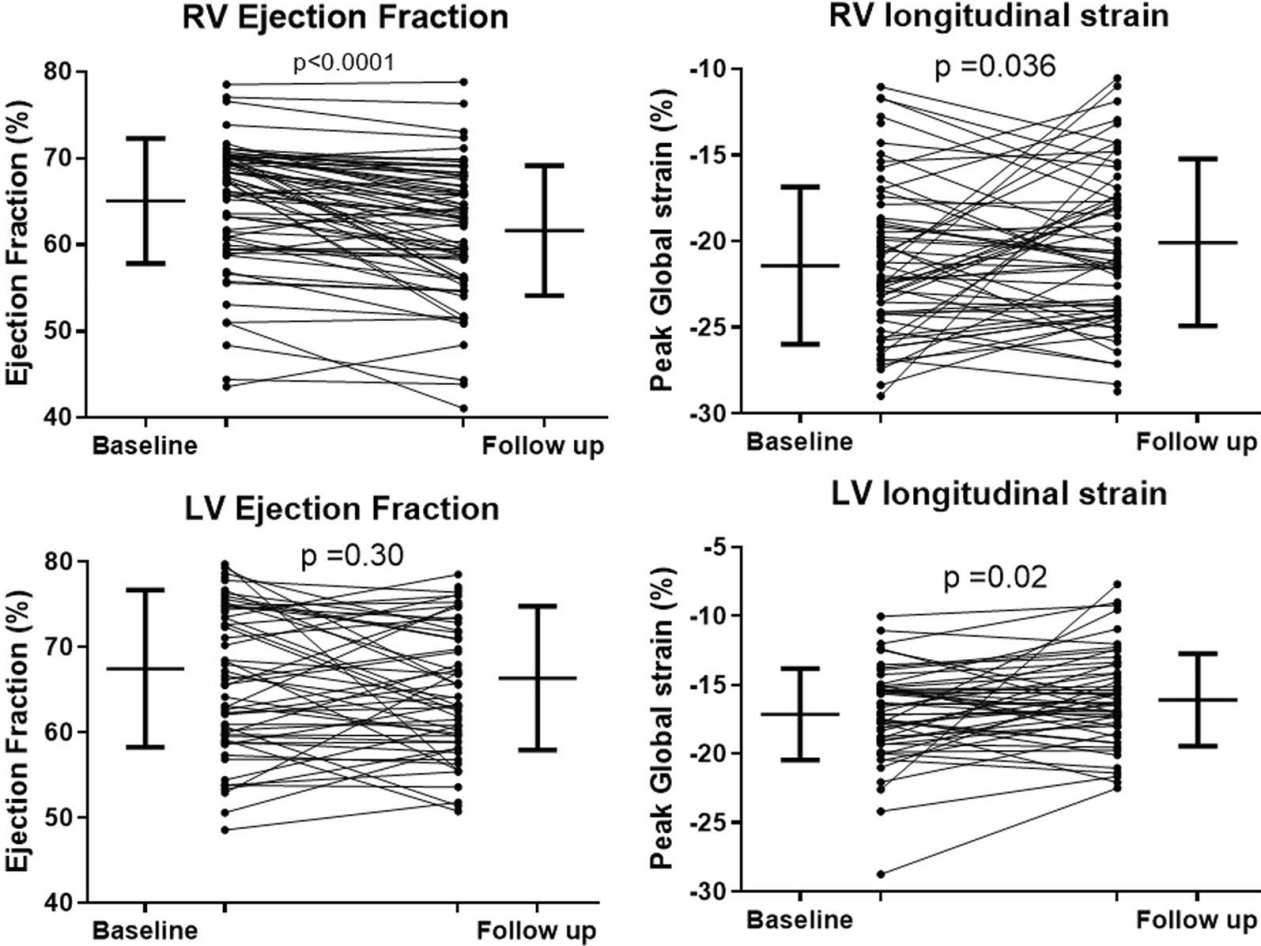
Dot plots for left ventricular (LV)/RV strain and LV ejection fraction (LVEF)/RV ejection fraction (RVEF) over time

In 63 patients (mean 44 ± 13 years, 76% male), serial CMR was undertaken at a median interval of 5.4 years (interquartile range 4.2–7.1 years) and results are shown in Table 2 and Fig. 3. The subgroup of patients who underwent follow-up CMR study had similar baseline clinical characteristics as the patients in the main HCM cohort. There was a small but significant decline in RVEF over time. There was also small but significant reduction in RV longitudinal strain, but no significant change was seen in circumferential and radial strains when compared to the baseline scan. RV mass was increased at the follow-up CMR.

**Table 2:** Follow-up CMR assessment of RV and LV function (n = 63)

CMR results, (± SD)	Baseline CMR	Follow-up CMR	P-value	Difference in mean (95% CI)
RVEDV (ml)	136 ± 35	130 ± 27	0.07	6.5 (− 0.4 to 13.5)
RVESV (ml)	48 ± 19	50 ± 15	0.20	− 1.8 (-4.9 to 1.3)
RVSV (ml)	89 ± 22	80 ± 18	0.001	8.4 (3.6 to 13.1)
RVEF (%)	65 ± 7	62 ± 7	< 0.001	3.5 (2.3 to 4.8)
RV mass (g)	39 ± 11	42 ± 11	0.02	− 3.0 (− 5.5 to − 0.5)
RV peak radial strain (%)	17 ± 7	18 ± 6	0.10	− 1.5 (− 3.2 to 0.3)
RV peak circumferential strain (%)	− 10 ± 4	− 11 ± 5	0.13	1.0 (− 0.3 to − 2.3)
RV peak longitudinal strain (%)	− 21 ± 5	− 20 ± 5	0.036	− 1.3 (− 2.4 to − 0.1)
LVEDV (ml)	137 ± 32	131 ± 27	0.04	5.8 (0.4 to 11.2)
LVESV (ml)	46 ± 20	45 ± 17	0.63	0.8 (− 2.6 to 4.3)
LVSV (ml)	92 ± 20	86 ± 18	0.02	5.0 (1.0 to 8.9)
LVEF (%)	68 ± 10	66 ± 8	0.30	0.8 (− 2.6 to 4.3)
LV mass (g)	155 ± 57	161 ± 63	0.21	− 5.7 (− 14.7 to 3.3)
LV maximal wall thickness	20 ± 5	20 ± 5	0.52	− 0.3 (− 1.8 to 1.1)
LV peak radial strain (%)	37 ± 11	37 ± 12	0.76	0.4 (− 2.1 to 2.8)
LV peak circumferential strain (%)	− 18 ± 3	− 17 ± 4	0.008	− 1.3 (− 2.2 to − 0.4)
LV peak longitudinal strain (%)	− 17 ± 3	− 16 ± 3	0.02	− 1.1 (− 2.1 to − 0.2)

### Predictors of clinical outcomes

During a median of 4.4 years of follow up (interquartile range 2.9–6.6 years), 5 patients were lost to follow up. 73 (24%) patients had incident arrhythmias including NSVT (n = 46, 16%) and AF (n = 27, 9%), including one patient who had both. Sixteen (5%) patients had a HF outcome and 7 (2%) patients had a stroke. Seven (2%) patients died, of which 2 deaths were attributed to a cardiovascular cause. Thirty patients (11%) had ICD implantation for primary prevention. There were no SCDs nor appropriate shocks delivered by defibrillators.

On univariate analysis, both LV global longitudinal strain and LVEF were predictive of NSVT and composite cardiac events on univariate analysis. Additionally, LV global longitudinal strain was also predictive of AF, HF progression and all-cause mortality. The association was lost on multivariable analysis after adjusting for the respective univariate predictors (Additional file 2: shown in supplementary material) for each outcomes.

In contrast, RVEF predicted the risk of new onset AF, NSVT, HF outcomes and composite cardiac events while RV global longitudinal strain predicted NSVT on univariate Cox proportional analysis, as shown in Table 3. On multivariable analyses, the association with NSVT and composite CV outcomes remained for RVEF, while the association with NSVT remained with RV global longitudinal strain, after adjusting for the respective univariate predictors. RV indexed mass was also predictive of HF outcomes and all-cause mortality on univariate analysis, but the effect was lost on multivariable analysis. Presence of RV LGE did not predict any events on univariate analysis. The c-index of these model; 0.69 (95% CI 0.60–0.78) for RVEF and NSVT endpoint, 0.70 (95% CI 0.63–0.77) for RVEF and composite endpoint, 0.55 (95% CI 0.43–0.58) for RV longitudinal strain and NSVT endpoint and 0.51 (95% CI 0.43–0.58) for RV longitudinal strain and composite endpoint.

**Table 3:** Univariate and multivariable cox regression showing association between RV function and clinical outcomes

	Univariate Cox regression		Multivariable Cox regression	
	HR (95% CI)	P-value	HR (95% CI)*	P-value*
RVEF				
Non-sustained ventricular tachycardia	1.08 (1.05–1.12)	< 0.001	1.10 (1.06–1.15)	< 0.001
Atrial fibrillation	1.06 (1.01–1.11)	0.017	1.04 (0.99–1.10)	0.08
Heart failure outcomes	1.07 (0.99–1.13)	0.037	0.99 (0.89–1.09)	0.77
Stroke	1.08 (0.98–1.19)	0.142		
All-cause mortality	1.08 (0.98–1.19)	0.109		
Composite cardiovascular events	1.07 (1.05–1.10)	< 0.001	1.07 (1.03–1.10)	< 0.001
RV global longitudinal strain				
Non-sustained ventricular tachycardia	1.06 (1.02–1.10)	0.003	1.05 (1.01–1.09)	0.029
Atrial fibrillation	1.02 (0.96–1.08)	0.49		
Heart failure outcomes	0.96 (0.87–1.05)	0.37		
Stroke	1.03 (0.97–1.10)	0.30		
All-cause mortality	0.96 (0.96–1.11)	0.71		
Composite cardiovascular events	1.03 (1.00–1.06)	0.097		

*Multivariable cox regression analysis is described in the Additional file 2: supplement

**Fig. 4:**
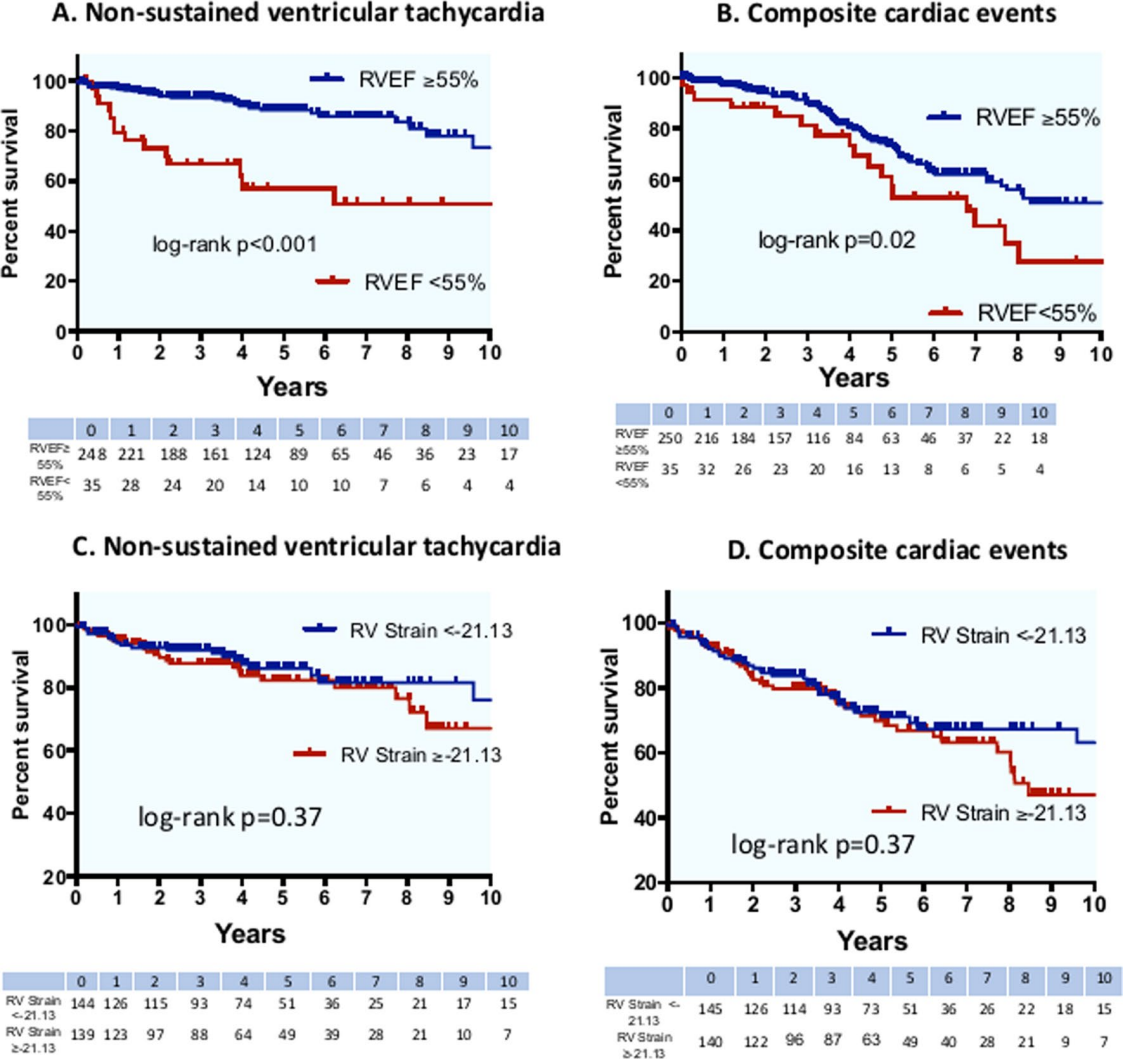
RVEF and RV strain (divided based on medians) and clinical outcomes. Kaplan–Meier curves showing associations between RV function (RVEF and RV strain) and **A** incident non-sustained ventricular tachycardia (NSVT), **B** composite cardiac events

In patients with impaired RV function (RVEF < 55%), there was a three-fold increase in NSVT (HR 3.42, 95% CI 1.81–6.44, P < 0.001) and a two-fold increase in composite cardiac events (HR 2.09, 95% CI 1.24–3.51, P = 0.006) on univariate analysis as shown in Fig. 4A and B. Additionally, RVEF < 55% was also predictive of all-cause mortality (HR 5.15, 95% CI 1.15–23.04, P = 0.03) but not HF outcomes or stroke. In contrast, RV longitudinal strain stratified based on median values did not predict NSVT or composite endpoints.

We further examined the prognostic role of RV function in determining composite events and NSVT in patients under 55 years old. RVEF remained predictive of both composite cardiac outcomes and NSVT in patients ≤ 55 years and above.

## Discussion

The current study shows three important findings. First, RV and LV strains were impaired in patients with HCM despite normal LVEF. There was a small reduction in RVEF, albeit within accepted normal limits. Second, whilst LVEF remained stable, both RVEF and RV strain, as well as LV strain parameters progressively declined in HCM over time. Third, RVEF was an independent predictors of adverse cardiovascular outcomes even after adjusted for conventional LV parameters such as LV wall thickness and LGE.

### RV function is impaired despite preserved LV function in hypertrophic cardiomyopathy

Whilst both morphological and functional changes of the LV have been well characterised in HCM, less is known about the RV function owing to difficulties encountered during assessment of the RV, arising from its complex geometry [20]. Two-dimensional echocardiography is the most widely used imaging modality for RV assessment, but remains limited due to dependence of image quality on operator experience and subject characteristics (body habitus and comorbidities). The lack of clear endocardial definition on echocardiography can also contribute to inaccuracies of RV chamber size estimation, wall thickness and function [20]. In the current study, we used CMR to assess the RV wall thickness, volumes and function in HCM. In contrast to echocardiography, CMR has been shown to be more accurate for the measurement of RV function, providing 3-dimensional coverage of the heart [13] and alleviating the need for geometric assumptions about shape. In line with this, we and others [21] found both measurements of RVEF and strain on CMR to be highly reproducible with good inter and intraobserver variability.

In the present study, we showed that despite normal LVEF, RV function, in particular RV strains in HCM patients were significantly reduced, with a small reduction in RVEF although this was still within the normal range, for an age- and sex-matched controls. We also showed that LV strain parameters were reduced in line with a previous study [22]. Our findings are in agreement with a smaller echocardiography study of 43 HCM which also reported subtle impairment in RV function [23], though the evidence in support of RV dysfunction has been conflicting in previous studies [9, 24]. Here, we also found no evidence that reduced RV function was secondary to increased pulmonary artery pressure, indicating that RV dysfunction is an intrinsic feature of HCM [25] and contesting the commonly accepted notion that RV failure may be an adaptive response to increased pulmonary pressures and LV dysfunction in HCM.

Although the mechanisms underlying RV dysfunction in HCM are unclear, previous studies have reported histological evidence of hypertrophy, myocyte disarray and fibrosis involving the RV of HCM patients [26]. Indeed, RV hypertrophy and fibrosis (in the form of LGE on CMR) have also been reported in other non-invasive imaging studies [6, 8, 9] suggesting that the cardiomyopathic process in HCM is likely to be more diffuse. Given that myocardial fibrosis and disarray may contribute to contractile abnormalities of the LV [27–29], it seems plausible that RV dysfunction in our patients may be the result of intrinsic myocardial disease [26, 30] caused by the sarcomeric mutant protein which would be found in all cardiomyocytes (involving both left and right ventricles).

Myocardial strain is a sensitive tool for the assessment of subclinical cardiac dysfunction on both echocardiogram and CMR [31]. Although RV strain is not as widely used as LV strain, we and others [21] have found RV strain to be highly reproducible using CMR tissue tracking. As with RVEF (although small reduction), RV longitudinal strain was impaired in HCM patients, a finding consistent with previous echocardiographic studies [32–34]. Additionally, we show that on CMR, RV circumferential and radial strain were also reduced in HCM patients. Interestingly, RV longitudinal strain was worse in those with more severe LV hypertrophy, implying that RV function is linked to the phenotypic severity of HCM [32]. Taken together, these data suggest that the comprehensive evaluation of RV function on CMR may be useful in detecting the extent of disease involvement in HCM despite normal LVEF.

### RV function in HCM declines over time

This study also examined the natural history of RV dysfunction on interval CMR scans in a subset of patients. We found a significant decrease in RVEF and longitudinal strain, but not circumferential or radial strain. Interestingly, the reduction in RVEF was not accompanied by a parallel reduction in LVEF, though LV global longitudinal strain did decrease over time. This would suggest that RVEF may be more sensitive than LVEF to disease progression [35]. We have shown that in HCM, myocardial fibrosis and LV hypertrophy can progress on serial CMR [36] and affects subtle markers of contractility such as global longitudinal strain. Based on these findings, we postulate that the reduction in RV function may also be due to an accumulating burden of pathology. Given that the RV is relatively thin-walled, changes in myocardial architecture (such as hypertrophy, disarray or fibrosis) could potentially alter myocardial contractility to a greater extent than that seen in the muscular LV. Whilst the qualitative and quantitative assessment of myocardial fibrosis/LGE in the RV could in theory help resolve this, detection of fibrosis in the RV is challenging and frequently confounded by partial volume effects of blood pool due to the thin-walled, trabeculated myocardium of the RV. The low frequency of focal RV LGE (3%) in our study and others [9] could additionally be reflective of these technical limitations as such, further development in acquisition strategies (high resolution imaging) may be required to improve the accuracy of fibrosis assessment in the RV. The impact of worsening LV stiffness and diastolic dysfunction also deserves further consideration [37] as increasing pulmonary vascular resistance is likely to increase the RV afterload and consequently function. In the present study serial imaging was only limited to CMR and not echocardiography and hence we remain limited in our ability to clarify delineate the specific cause of progressive RV dysfunction.

### RV function predicts adverse cardiovascular outcomes

In this study, clinical follow up of 290 HCM patients was undertaken over a median interval of 4.4 years. Previous studies have found RV hypertrophy to be prognostically important. Here, although maximum RV wall thickness was a determinant of HF outcomes on univariate analysis, there was no association seen with ventricular arrythmias. Additionally, in our study the relationship between RV wall thickness and HF progression was lost after adjusting for RV function and other univariate predictors. The presence of LGE in the RV also did not associate with clinical outcomes and is likely to reflect the reduced power of this study owing to the low frequency of RV LGE. On the other hand, reduced RVEF was predictive of NSVT and composite cardiovascular events (NSVT, HF outcomes and cardiovascular death), while RV global longitudinal strain was predictive of NSVT even after adjusting for confounding variables (although its diagnostic performance in predicting such events remain limited). These findings are consistent with a recent study by Shah et al. [7] which showed an increased risk of cardiovascular mortality in HCM patients with moderate RV systolic dysfunction (RVEF < 45%) and evidence of LV impairment. Our study extends these findings as we show that even in cases with mild RV dysfunction (RVEF < 55%) and preserved LVEF, RV function independently predicted poor clinical outcomes.

Another interesting observation was the association of RVEF with NSVT and composite cardiovascular events in those under the age of 55. Ho et al. [38] have similarly noted that younger patients with HCM tended to have higher risk of HF and life-threatening arrhythmias compared to age matched healthy population. As CMR is increasingly performed for clarification of anatomy and risk stratification with LGE imaging [3], extending the assessment to involve RV function and strain may help refine risk prediction particularly in younger HCM patients and serve as a guide for closer surveillance.

### Study limitations

The limitations of this study follow from its observational, single-centre study design. The population studied were at low risk for SCD and some of the endpoints (NSVT and AF) chosen, although clinically relevant, were soft. Furthermore, details about the number of ventricular triplets of NSVT, which on its own is a soft end point were not available. The echocardiographic analysis of the RV was performed as per clinical need, therefore targeted RV acquisition may have been suboptimal, particularly early on in the enrolment period before the publication of American Society of Echocardiography (ASE) guidelines for evaluation of the right heart in 2010 [39]. The lack of serial echocardiography in the longitudinal assessment limits our ability to draw definitive conclusions on cause for progressive RV dysfunction. Similarly, diastolic functional parameters, in line with contemporary guidelines, were available only for those patients who underwent CMR scans after 2010 [40]. Follow up CMR was undertaken in only 63 patients as a part of a natural history sub-study (funding limited) specifically designed to examine differences in left and right ventricular parameters over time. As such, this may not have been sufficiently powered and may be unrepresentative of the baseline cohort to examine the prognostic value of progressive RV dysfunction. RV afterload was not formally investigated with right heart catheterisation, but echocardiography provided an estimation of pulmonary arterial pressure. The prevalence of visible LGE in the RV was low, in line with a previous study [9], and may be underestimated due to partial volume effects of blood. Finally, our results may be sustained by an exceptional reproducibility in RVEF measurement, better than previously reported in 2004 [41].

## Conclusions

In HCM, RV function, including RV strain, and LV strain may decline over time despite stable LVEF. Reduced RVEF is associated with NSVT and composite cardiovascular events, while global longitudinal strain is associated with NSVT. RV function may be more sensitive than LV functional parameters for disease prognostication at an early stage. These findings warrant large-scale assessment of RV parameters as risk predictors in HCM.

## Supplementary Information

**Additional file1 .**

**Additional file2 .**

## Data Availability

Dataset used in this study are available from the corresponding author on reasonable request.

## Abbreviations

AF: Atrial fibrillation
BMI: Body mass index
CMR: Cardiovascular magnetic resonance
FT: Feature tracking
HCM: Hypertrophic cardiomyopathy
HF: Heart failure
ICD: Implantable cardiodefibrillator
LA: Left atrium/left atrial
LGE: Late gadolinium enhancement
LV: Left ventricle/left ventricular
LVEF: Left ventricular ejection fraction
NSVT: Non-sustained ventricular tachycardia
NYHA: New York Heart Association
RV: Right ventricle/right ventricular
RVEF: Right ventricular ejection fraction
RVFWS: Right ventricular free wall strain
SAx: Short axis
SCD: Sudden cardiac death
