# Supplementary material for: Right ventricular function declines prior to left ventricular ejection fraction in hypertrophic cardiomyopathy
Source: J Cardiovasc Magn Reson. 2022 Jun 13;24:36. doi: 10.1186/s12968-022-00868-y (PMC9190122; doi:10.1186/s12968-022-00868-y)
Supplement: Supplementary file 2 — Additional file2 . [file 12968_2022_868_MOESM2_ESM.docx]

**Inter and intra- observer measurement for right ventricular function**

The interobserver variability was good for all measurements of right ventricular (RV) function with intra-class correlation coefficients (ICC) of 0.95 (95% CI 0.86-0.98) for RV circumferential strain, 0.92 (95% CI 0.82-0.96) for longitudinal strain and 0.90 (95% CI 0.79-0.95) for radial strain. Intra-observer ICC’s were also excellent for RV ejection fraction (RVEF) – 0.96 (95% CI 0.86-0.99), circumferential strain 0.97 (0.9-0.99), radial strain 0.94 (95% CI 0.79-0.99) and longitudinal strain 0.96 (0.86-0.99).

**Sample size calculation for the study**

The sample size estimated for this study was based on the cumulative event rate for the combined cardiovascular endpoint which included non-sustained ventricular tachycardia (NSVT), sudden cardiac death or aborted sudden cardiac, all cause cardiovascular death, atrial fibrillation, stroke or embolic events, heart failure outcomes. In a previous study by Ho *et al* (1), the cumulative incidence of composite cardiac outcomes in HCM patients ranged from 30 - 77% depending on age. Using a conservative composite event rate 40%, power of 80%, Type 1 error rate of 5%, a minimum sample size of 256 HCM patients was required to predict a 2-fold increased risk of the composite cardiovascular events in those with impaired RV function given a variability of 0.40.

**Multivariable analyses for LV ejection fraction and LV longitudinal strain**

The variables included in each of the multivariate models were univariate predictors for each of the pre-specified outcome. In places where two variables were highly correlated (eg, LV mass versus wall thickness), the variable with the highest hazard ratio was used and adjusted for in the multivariable model to avoid for collinearity (Tables 1-4). In the case of NSVT, we adjusted for age, left atrial (LA diameter), syncope, medication use, right ventricular (RV) mass, RVEF, left ventricular (LV) maximum wall thickness and late gadolinium enhancement (LGE) presence. For atrial fibrillation, the multivariable model was adjusted for age and RVEF. For heart failure outcomes, we adjusted for age, gender, body mass index, NYHA class, RV systolic volume, RV mass index, RV circumferential strain, LV end diastolic volume, LV maximum wall thickness, LGE . With regards to stroke, we adjusted for age and hypertension, RV circumferential strain, LGE mass, crista supraventricularis. The multivariable model for all-cause mortality was adjusted for age, RV mass index, LV mass index and LGE mass. For composite cardiovascular events, the model was adjusted for univariate predictors which included age, BMI, LA diameter, NYHA class, medication use, maximum LV wall thickness , LGE presence, family history of sudden cardiac death, RVEF, RVSV, LV Mass index, LGE mass and crista supraventricularis.

**Table 1. Univariable Cox Regression for NSVT in HCM**

|  | **Univariate Cox Regression** | | | |
| --- | --- | --- | --- | --- |
|  | **HR** | **(95% CI)** | **P-value** |  |
| Age | 1.02 | (1.00-1.05) | 0.057 |  |
| Gender | 1.29 | (0.62-2.69) | 0.491 |  |
| Body Mass Index | 1.03 | (0.97-1.10) | 0.337 |  |
| Smoking status | 0.89 | (0.50-1.58) | 0.687 |  |
| Hypertension | 1.13 | (0.61-2.10) | 0.694 |  |
| Diabetes | 1.49 | (0.59-3.77) | 0.406 |  |
| Family history of sudden cardiac death | 0.21 | (0.03-1.53) | 0.124 |  |
| Syncope | 2.20 | (1.14-4.27) | 0.019 |  |
| Left atrial diameter in LV outflow tract view (mm) | 1.06 | (1.01-1.11) | 0.018 |  |
| LV outflow tract obstruction gradient >30 | 1.73 | (0.42-7.19) | 0.451 |  |
| NYHA class (I,II,III,IV) | 1.52 | (0.99-2.33) | 0.054 |  |
| Medication use | 1.56 | (1.07-2.28) | 0.021 |  |
| RV end diastolic volume (ml) | 1.00 | (0.99-1.00) | 0.463 |  |
| RV end systolic volume (ml) | 1.01 | (1.00-1.02) | 0.167 |  |
| RV systolic volume (ml) | 0.99 | (0.98-1.00) | 0.029 |  |
| RV ejection fraction (%) | 1.08 | (1.05-1.12) | <0.000 |  |
| RV Mass (g) | 1.03 | (1.00-1.06) | 0.022 |  |
| RV Mass index (g/m^2^) | 1.06 | (1.00-1.13) | 0.055 |  |
| Maximum RV wall thickness (mm) | 0.99 | (0.96-1.03) | 0.676 |  |
| RV hypertrophy (0/1) | 0.52 | (0.22-1.23) | 0.137 |  |
| Late gadolinium enhancement of RV free wall (0/1) | 1.07 | (0.15-7.79) | 0.948 |  |
| RV radial strain (%) | 0.99 | (0.95-1.04) | 0.841 |  |
| RV circumferential strain (%) | 1.02 | (0.95-1.10) | 0.573 |  |
| RV longitudinal strain (%) | 1.06 | (1.02-1.10) | 0.003 |  |
| LV end diastolic volume (ml) | 1.00 | (0.99-1.01) | 0.538 |  |
| LV end systolic volume (ml) | 1.01 | (0.99-1.02) | 0.516 |  |
| LV stroke volume (ml) | 0.99 | (0.98-1.00) | 0.203 |  |
| LV ejection fraction (%) | 1.05 | (1.00-1.10) | 0.035 |  |
| LV Mass (g) | 1.00 | (1.00-1.01) | 0.114 |  |
| LV Mass index (g/mm^2^) | 1.01 | (1.00-1.02) | 0.188 |  |
| Max LV Wall Thickness (mm) | 1.11 | (1.05-1.17) | <0.001 |  |
| LV radial strain (%) | 1.00 | (0.97-1.03) | 0.883 |  |
| LV circumferential strain (%) | 1.00 | (0.93-1.08) | 0.940 |  |
| LV longitudinal strain (%) | 1.09 | (1.01-1.17) | 0.033 |  |
| Late gadolinium enhancement mass (g) | 1.01 | (1.00-1.03) | 0.118 |  |
| Late gadolinium enhancement (0/1) | 2.96 | (1.06-8.29) | 0.039 |  |
| Crista supraventricularis (0/1) | 1.28 | (0.50-3.26) | 0.604 |  |

**Table 2. Univariable Cox Regression for Atrial Fibrillation in HCM**

|  | **Univariate Cox Regression** | | | |
| --- | --- | --- | --- | --- |
|  | **HR** | **(95% CI)** | **P-value** |  |
| Age | 1.06 | (1.03-1.10) | <0.0001 |  |
| Gender | 1.17 | (0.47-2.90) | 0.742 |  |
| Body Mass Index | 1.01 | (0.93-1.09) | 0.880 |  |
| Smoking status | 0.69 | (0.29-1.63) | 0.395 |  |
| Hypertension | 1.42 | (0.67-3.01) | 0.367 |  |
| Diabetes | 0.44 | (0.06-3.25) | 0.421 |  |
| Family history of sudden cardiac death | 0.37 | (0.05-2.72) | 0.328 |  |
| Syncope | 0.78 | (0.24-2.61) | 0.691 |  |
| Left atrial diameter in LV outflow tract view (mm) | 1.04 | (0.98-1.11) | 0.207 |  |
| LV outflow tract obstruction gradient >30 | 0.05 | (0.00-1144) | 0.554 |  |
| NYHA class (I,II,III,IV) | 1.31 | (0.72-2.38) | 0.382 |  |
| Medication use | 1.57 | (0.96-2.55) | 0.072 |  |
| RV end diastolic volume (ml) | 1.00 | (0.99-1.01) | 0.697 |  |
| RV end systolic volume (ml) | 1.01 | (0.99-1.02) | 0.457 |  |
| RV systolic volume (ml) | 0.99 | (0.98-1.01) | 0.246 |  |
| RV ejection fraction (%) | 1.06 | (1.01-1.11) | 0.016 |  |
| RV Mass (g) | 1.00 | (0.97-1.04) | 0.981 |  |
| RV Mass index (g/m^2^) | 0.98 | (0.90-1.07) | 0.715 |  |
| Maximum RV wall thickness (mm) | 0.99 | (0.93-1.05) | 0.761 |  |
| RV hypertrophy (0/1) | 1.71 | (0.77-3.80) | 0.191 |  |
| Late gadolinium enhancement of RV free wall (0/1) | 1.92 | (0.26-14.2) | 0.525 |  |
| RV radial strain (%) | 1.04 | (0.98-1.10) | 0.171 |  |
| RV circumferential strain (%) | 0.99 | (0.95-1.03) | 0.585 |  |
| RV longitudinal strain (%) | 1.02 | (0.96-1.08) | 0.491 |  |
| LV end diastolic volume (ml) | 1.00 | (0.99-1.01) | 0.592 |  |
| LV end systolic volume (ml) | 1.00 | (0.97-1.02) | 0.768 |  |
| LV stroke volume (ml) | 1.00 | (0.98-1.01) | 0.560 |  |
| LV ejection fraction (%) | 1.00 | (0.95-1.06) | 0.903 |  |
| LV Mass (g) | 1.00 | (1.00-1.01) | 0.156 |  |
| LV Mass index (g/mm^2^) | 1.01 | (1.00-1.03) | 0.089 |  |
| Max LV Wall Thickness (mm) | 1.06 | (0.98-1.14) | 0.126 |  |
| LV radial strain (%) | 0.99 | (0.96-1.03) | 0.573 |  |
| LV circumferential strain (%) | 1.05 | (0.96-1.15) | 0.323 |  |
| LV longitudinal strain (%) | 1.15 | (1.05-1.27) | 0.004 |  |
| Late gadolinium enhancement mass (g) | 1.02 | (1.00-1.04) | 0.102 |  |
| Late gadolinium enhancement (0/1) | 3.67 | (0.87-15.5) | 0.078 |  |
| Crista supraventricularis (0/1) | 2.63 | (0.99-7.03) | 0.054 |  |

**Table 3. Univariable Cox Regression for Heart failure outcomes in HCM**

|  | **Univariate Cox Regression** | | | |
| --- | --- | --- | --- | --- |
|  | **HR** | **(95% CI)** | **P-value** |  |
| Age | 1.05 | (1.01-1.10) | 0.019 |  |
| Gender | 0.36 | (0.13-1.00) | 0.049 |  |
| Body Mass Index | 1.13 | (1.03-1.24) | 0.007 |  |
| Smoking status | 0.91 | (0.34-2.48) | 0.856 |  |
| Hypertension | 1.21 | (0.42-3.45) | 0.728 |  |
| Diabetes | 2.78 | (0.78-9.88) | 0.114 |  |
| Family history of sudden cardiac death | 0.04 | (0.00-99.1) | 0.425 |  |
| Syncope | 1.37 | (0.39-4.85) | 0.628 |  |
| Left atrial diameter in LV outflow tract view (mm) | 1.05 | (0.97-1.14) | 0.217 |  |
| LV outflow tract obstruction gradient >30 | 0.05 | (0.00-50995) | 0.667 |  |
| NYHA class (I,II,III,IV) | 8.33 | (4.55-15.3) | <0.0001 |  |
| Medication use | 1.35 | (0.70-2.61) | 0.377 |  |
| RV end diastolic volume (ml) | 0.99 | (0.97-1.02) | 0.517 |  |
| RV end systolic volume (ml) | 0.97 | (0.95-0.99) | 0.012 |  |
| RV systolic volume (ml) | 1.07 | (1.00-1.14) | 0.037 |  |
| RV ejection fraction (%) | 0.98 | (0.94-1.03) | 0.465 |  |
| RV Mass (g) | 0.97 | (0.87-1.09) | 0.625 |  |
| RV Mass index (g/m^2^) | 1.01 | (1.00-1.01) | 0.033 |  |
| Maximum RV wall thickness (mm) | 1.31 | (0.42-4.12) | 0.643 |  |
| RV hypertrophy (0/1) | 0.05 | (0.00-175700) | 0.694 |  |
| Late gadolinium enhancement of RV free wall (0/1) | 0.97 | (0.89-1.06) | 0.480 |  |
| RV radial strain (%) | 1.04 | (0.94-1.12) | 0.345 |  |
| RV circumferential strain (%) | 1.00 | (0.94-1.10) | 0.972 |  |
| RV longitudinal strain (%) | 0.96 | (0.87-1.05) | 0.373 |  |
| LV end diastolic volume (ml) | 0.97 | (0.95-1.00) | 0.019 |  |
| LV end systolic volume (ml) | 0.95 | (0.87-1.03) | 0.197 |  |
| LV stroke volume (ml) | 1.00 | (0.99-1.01) | 0.532 |  |
| LV ejection fraction (%) | 1.06 | (0.97-1.15) | 0.196 |  |
| LV Mass (g) | 1.03 | (0.93-1.15) | 0.593 |  |
| LV Mass index (g/mm^2^) | 0.96 | (0.91-1.01) | 0.136 |  |
| Max LV Wall Thickness (mm) | 1.16 | (1.02-1.33) | 0.029 |  |
| LV radial strain (%) | 1.18 | (1.06-1.33) | 0.004 |  |
| LV circumferential strain (%) | 1.01 | (0.98-1.04) | 0.494 |  |
| LV longitudinal strain (%) | 1.77 | (0.40-7.90) | 0.456 |  |
| Late gadolinium enhancement mass (g) | 0.76 | (0.10-5.81) | 0.792 |  |
| Late gadolinium enhancement (0/1) | 1.05 | (1.01-1.10) | 0.019 |  |
| Crista supraventricularis (0/1) | 0.36 | (0.13-1.00) | 0.049 |  |

**Table 4. Univariable Cox Regression for Stroke in HCM**

|  | **Univariate Cox Regression** | | | | |
| --- | --- | --- | --- | --- | --- |
|  | **HR** | | **(95% CI)** | **P-value** |  |
| Age | | 1.08 | (1.01-1.17) | 0.028 |  |
| Gender | | 0.54 | (0.10-3.07) | 0.488 |  |
| Body Mass Index | | 0.98 | (0.82-1.18) | 0.855 |  |
| Smoking status | | 0.08 | (0.00-86.97) | 0.477 |  |
| Hypertension | | 5.12 | (1.10-23.85) | 0.038 |  |
| Diabetes | | 2.29 | (0.27-19.66) | 0.450 |  |
| Family history of sudden cardiac death | | 0.04 | (0.00-13077) | 0.626 |  |
| Syncope | | 1.22 | (0.14-10.58) | 0.854 |  |
| Left atrial diameter in LV outflow tract view (mm) | | 1.08 | (0.95-1.22) | 0.233 |  |
| LV outflow tract obstruction gradient >30 | | 0.05 | (0.00-4670207k) | 0.813 |  |
| NYHA class (I,II,III,IV) | | 1.84 | (0.61-5.54) | 0.277 |  |
| Medication use | | 2.98 | (0.98-9.12) | 0.055 |  |
| RV end diastolic volume (ml) | | 0.99 | (0.97-1.01) | 0.373 |  |
| RV end systolic volume (ml) | | 1.00 | (0.96-1.04) | 0.953 |  |
| RV systolic volume (ml) | | 0.98 | (0.95-1.01) | 0.183 |  |
| RV ejection fraction (%) | | 1.08 | (0.98-1.19) | 0.142 |  |
| RV Mass (g) | | 1.03 | (0.96-1.10) | 0.408 |  |
| RV Mass index (g/m^2^) | | 1.09 | (0.93-1.27) | 0.304 |  |
| Maximum RV wall thickness (mm) | | 1.00 | (0.95-1.05) | 0.913 |  |
| RV hypertrophy (0/1) | | 0.76 | (0.09-6.56) | 0.805 |  |
| Late gadolinium enhancement of RV free wall (0/1) | | 0.05 | (0.00-6993654k) | 0.817 |  |
| RV radial strain (%) | | 1.06 | (0.95-1.18) | 0.321 |  |
| RV circumferential strain (%) | | 0.99 | (0.92-1.05) | 0.673 |  |
| RV longitudinal strain (%) | | 0.80 | (0.66-1.97) | 0.022 |  |
| LV end diastolic volume (ml) | | 0.99 | (0.96-1.01) | 0.309 |  |
| LV end systolic volume (ml) | | 0.99 | (0.94-1.05) | 0.729 |  |
| LV stroke volume (ml) | | 0.98 | (0.95-1.01) | 0.231 |  |
| LV ejection fraction (%) | | 1.05 | (0.92-1.20) | 0.453 |  |
| LV Mass (g) | | 1.01 | (1.00-1.02) | 0.191 |  |
| LV Mass index (g/mm^2^) | | 1.02 | (0.99-1.05) | 0.144 |  |
| Max LV Wall Thickness (mm) | | 1.12 | (0.96-1.30) | 0.140 |  |
| LV radial strain (%) | | 1.01 | (0.94-1.08) | 0.883 |  |
| LV circumferential strain (%) | | 1.03 | (0.84-1.25) | 0.798 |  |
| LV longitudinal strain (%) | | 1.20 | (1.00-1.44) | 0.056 |  |
| Late gadolinium enhancement mass (g) | | 1.03 | (1.00-1.07) | 0.031 |  |
| Late gadolinium enhancement (0/1) | | 0.62 | (0.11-3.44) | 0.580 |  |
| Crista supraventricularis (0/1) | | 6.48 | (1.08-38.83) | 0.041 |  |

**Table 4. Univariable Cox Regression for All-Cause Mortality in HCM**

|  | **Univariate Cox Regression** | | | |
| --- | --- | --- | --- | --- |
|  | **HR** | **(95% CI)** | **P-value** |  |
| Age | 1.13 | (1.05-1.21) | 0.002 |  |
| Gender | 0.77 | (0.15-3.99) | 0.757 |  |
| Body Mass Index | 0.90 | (0.75-1.08) | 0.270 |  |
| Smoking status | 0.68 | (0.12-3.87) | 0.665 |  |
| Hypertension | 2.88 | (0.73-11.35) | 0.130 |  |
| Diabetes | 1.71 | (0.21-14.23) | 0.621 |  |
| Family history of sudden cardiac death | 0.04 | (0.00-3899) | 0.589 |  |
| Syncope | 4.05 | (0.91-18.14) | 0.067 |  |
| Left atrial diameter in LV outflow tract view (mm) | 1.09 | (0.97-1.22) | 0.158 |  |
| LV outflow tract obstruction gradient >30 | 0.05 | (0.00-37129k) | 0.770 |  |
| NYHA class (I,II,III,IV) | 1.04 | (0.29-3.72) | 0.955 |  |
| Medication use | 1.16 | (0.44-3.09) | 0.763 |  |
| RV end diastolic volume (ml) | 0.99 | (0.97-1.01) | 0.309 |  |
| RV end systolic volume (ml) | 1.00 | (0.96-1.03) | 0.820 |  |
| RV systolic volume (ml) | 0.98 | (0.95-1.01) | 0.164 |  |
| RV ejection fraction (%) | 1.08 | (0.98-1.19) | 0.109 |  |
| RV Mass (g) | 1.05 | (0.99-1.12) | 0.109 |  |
| RV Mass index (g/m^2^) | 1.18 | (1.03-1.34) | 0.018 |  |
| Maximum RV wall thickness (mm) | 1.00 | (0.97-1.03) | 0.937 |  |
| RV hypertrophy (0/1) | 0.61 | (0.07-5.06) | 0.645 |  |
| Late gadolinium enhancement of RV free wall (0/1) | 0.05 | (0.00-125455k) | 0.784 |  |
| RV radial strain (%) | 0.97 | (0.87-1.10) | 0.655 |  |
| RV circumferential strain (%) | 1.05 | (0.87-1.26) | 0.594 |  |
| RV longitudinal strain (%) | 0.98 | (0.86-1.11) | 0.710 |  |
| LV end diastolic volume (ml) | 0.98 | (0.96-1.00) | 0.101 |  |
| LV end systolic volume (ml) | 0.96 | (0.91-1.02) | 0.177 |  |
| LV stroke volume (ml) | 0.97 | (0.94-1.01) | 0.127 |  |
| LV ejection fraction (%) | 1.01 | (0.90-1.13) | 0.927 |  |
| LV Mass (g) | 1.01 | (1.00-1.02) | 0.130 |  |
| LV Mass index (g/mm^2^) | 1.03 | (1.00-1.05) | 0.036 |  |
| Max LV Wall Thickness (mm) | 1.13 | (0.99-1.29) | 0.069 |  |
| LV radial strain (%) | 0.98 | (0.92-1.05) | 0.606 |  |
| LV circumferential strain (%) | 1.11 | (0.93-1.34) | 0.260 |  |
| LV longitudinal strain (%) | 1.22 | (1.03-1.44) | 0.022 |  |
| Late gadolinium enhancement mass (g) | 1.03 | (1.00-1.06) | 0.026 |  |
| Late gadolinium enhancement (0/1) | 1.64 | (0.20-13.74) | 0.650 |  |
| Crista supraventricularis (0/1) | 1.82 | (0.22-15.18) | 0.579 |  |

**Table 4. Univariable Cox Regression for Composite Cardiovascular Events in HCM**

|  | **Univariate Cox Regression** | | | |
| --- | --- | --- | --- | --- |
|  | **HR** | **(95% CI)** | **P-value** |  |
| Age | 1.04 | (1.02-1.06) | <0.0001 |  |
| Gender | 0.91 | (0.56-1.48) | 0.690 |  |
| Body Mass Index | 1.05 | (1.01-1.10) | 0.020 |  |
| Smoking status | 0.74 | (0.46-1.17) | 0.196 |  |
| Hypertension | 1.31 | (0.85-2.03) | 0.225 |  |
| Diabetes | 1.53 | (0.77-3.06) | 0.230 |  |
| Family history of sudden cardiac death | 0.23 | (0.06-0.92) | 0.038 |  |
| Syncope | 1.54 | (0.91-2.63) | 0.111 |  |
| Left atrial diameter in LV outflow tract view (mm) | 1.06 | (1.03-1.10) | <0.001 |  |
| LV outflow tract obstruction gradient >30 | 0.85 | (0.21-3.48) | 0.824 |  |
| NYHA class (I,II,III,IV) | 2.21 | (1.67-2.93) | <0.001 |  |
| Medication use | 1.57 | (1.19-2.07) | 0.001 |  |
| RV end diastolic volume (ml) | 1.00 | (0.99-1.00) | 0.200 |  |
| RV end systolic volume (ml) | 1.01 | (1.00-1.02) | 0.219 |  |
| RV systolic volume (ml) | 0.99 | (0.98-1.00) | 0.003 |  |
| RV ejection fraction (%) | 1.07 | (1.05-1.10) | <0.001 |  |
| RV Mass (g) | 1.02 | (1.00-1.04) | 0.101 |  |
| RV Mass index (g/m^2^) | 1.03 | (0.98-1.08) | 0.219 |  |
| Maximum RV wall thickness (mm) | 1.00 | (1.00-1.01) | 0.619 |  |
| RV hypertrophy (0/1) | 0.94 | (0.56-1.59) | 0.820 |  |
| Late gadolinium enhancement of RV free wall (0/1) | 1.15 | (0.28-4.70) | 0.843 |  |
| RV radial strain (%) | 1.02 | (0.99-1.06) | 0.211 |  |
| RV circumferential strain (%) | 0.99 | (0.97-1.03) | 0.973 |  |
| RV longitudinal strain (%) | 1.03 | (0.99-1.06) | 0.097 |  |
| LV end diastolic volume (ml) | 1.00 | (0.99-1.00) | 0.232 |  |
| LV end systolic volume (ml) | 1.00 | (0.99-1.02) | 0.764 |  |
| LV stroke volume (ml) | 0.96 | (0.93-1.00) | 0.033 |  |
| LV ejection fraction (%) | 1.04 | (1.00-1.07) | 0.033 |  |
| LV Mass (g) | 1.01 | (1.00-1.02) | 0.036 |  |
| LV Mass index (g/mm^2^) | 1.07 | (1.03-1.12) | 0.001 |  |
| Max LV Wall Thickness (mm) | 0.99 | (0.97-1.01) | 0.359 |  |
| LV radial strain (%) | 1.04 | (0.99-1.09) | 0.166 |  |
| LV circumferential strain (%) | 1.12 | (1.06-1.18) | <0.001 |  |
| LV longitudinal strain (%) | 1.01 | (1.00-1.03) | 0.021 |  |
| Late gadolinium enhancement mass (g) | 2.26 | (1.16-4.39) | 0.016 |  |
| Late gadolinium enhancement (0/1) | 1.43 | (0.74-2.78) | 0.290 |  |
| Crista supraventricularis (0/1) | 1.04 | (1.02-1.06) | <0.001 |  |

**Table 5. Univariable and multivariable cox regression showing association between left ventricular function and clinical outcomes**

|  | **Univariable Cox Regression** | | | **Multivariable Cox Regression** | | |
| --- | --- | --- | --- | --- | --- | --- |
|  | **HR (95% CI)** | **p-value** | **HR (95% CI)*** | | **p-value*** |  |
| **Left ventricular ejection fraction** |  |  |  | |  |  |
| Non-sustained ventricular tachycardia | 1.05 (1.00 -1.09) | 0.04 | 1.00 (0.94-1.06) | | 0.90 |  |
| Atrial fibrillation | 1.00 (0.95-1.06) | 0.90 |  | |  |  |
| Hospitalisation for heart failure | 1.06 (0.97-1.15) | 0.20 |  | |  |  |
| Cerebral ischaemic attack | 1.05 (0.92-1.20) | 0.45 |  | |  |  |
| All-cause mortality | 1.01 (0.90-1.13) | 0.93 |  | |  |  |
| Composite cardiovascular events | 1.04 (1.00-1.07) | 0.03 | 0.99 (0.94-1.05) | | 0.78 |  |
| **Left ventricular longitudinal strain** |  |  |  | |  |  |
| Non-sustained ventricular tachycardia | 1.09 (1. 01-1.17) | 0.03 | 0.95 (0.87-1.04) | | 0.30 |  |
| Atrial fibrillation | 1.15 (1.05-1.27) | 0.004 | 1.13 (1.02-1.25) | | 0.02 |  |
| Hospitalisation for heart failure | 1.18 (1.06-1.33) | 0.004 | 1.05 (0.87 -1.27) | | 0.62 |  |
| Cerebral ischaemic attack | 1.20 (1.00-1.44) | 0.06 |  | |  |  |
| All-cause mortality | 1.22 (1.03-1.44) | 0.02 | 0.97 (0.72-1.30) | | 0.88 |  |
| Composite cardiovascular events | 1.12 (1.06-1.18) | <0.0001 | 1.00 (0.93-1.07) | | 0.84 |  |

*multivariable analysis was adjusted univariable predictors of each outcomes

**Reference:**

1. Ho CY, Day SM, Ashley EA, Michels M, Pereira AC, Jacoby D, et al. Genotype and Lifetime Burden of Disease in Hypertrophic Cardiomyopathy: Insights from the Sarcomeric Human Cardiomyopathy Registry (SHaRe). Circulation. 2018;138(14):1387-98.
